# Effect of Co-Administration of Curcumin with Amlodipine in Hypertension

**DOI:** 10.3390/nu13082797

**Published:** 2021-08-15

**Authors:** Somin Lee, Cheolmin Jo, Ho-Young Choi, Kyungjin Lee

**Affiliations:** 1 Department of Biomedical Science and Technology, Graduate School, Kyung Hee University, Seoul 02447, Korea; sominleee@naver.com; 2 Department of Herbal Pharmacology, Graduate School, Kyung Hee University, Seoul 02447, Korea; chocm456@naver.com; 3 Department of Herbal Pharmacology, College of Korean Medicine, Kyung Hee University, Seoul 02447, Korea; hychoi@khu.ac.kr

**Keywords:** curcumin, amlodipine, co-administration, hypertension

## Abstract

Curcumin, a curcuminoid known as the main bioactive compound of turmeric, is used in foods, cosmetics, and pharmaceutical products. Amlodipine is a general antihypertensive drug used in combination with various other antihypertensive agents. To date, no studies have examined the effects of the co-administration of amlodipine with curcumin. In this study, the vasodilatory effects of curcumin, amlodipine, and the co-administration of curcumin with amlodipine on isolated rat aortic rings pre-contracted with phenylephrine were evaluated, and the hypotensive effects were evaluated using the tail cuff method. To measure blood pressure, male spontaneously hypertensive rats were divided into four groups, each containing six rats, as follows: amlodipine 1 mg/kg alone treated, amlodipine 1 mg/kg with curcumin 30 mg/kg treated, amlodipine 1 mg/kg with curcumin 100 mg/kg treated, and amlodipine 1 mg/kg with curcumin 300 mg/kg treated groups. Amlodipine and curcumin were intraperitoneally injected, and systolic blood pressure (SBP) and diastolic blood pressure (DBP) were measured at 1, 2, 4, and 8 h after administration. The combined administration of curcumin and amlodipine induced a stronger vasorelaxant effect than amlodipine alone. However, co-administration did not significantly lower SBP and DBP compared to the single administration of amlodipine. The results of this study suggest that hypertensive patients taking amlodipine can consume curcumin or turmeric for food or other medical purposes without inhibiting the blood pressure-lowering effect of amlodipine.

## 1. Introduction

Curcumin is a polyphenolic component of *Curcuma longa* L. (Zingiberaceae), widely known as turmeric, that is a bright yellow powder. Curcuminoids, known as the main biologically active compounds of turmeric, consist of curcumin, demethoxycurcumin, and bisdemethoxycurcumin, which only differ in the presence of one or two methoxy groups. Curcumin accounts for 75–80% of curcuminoids [1]. Curcumin has antioxidant [2,3], anti-inflammatory [3,4,5,6,7,8], and anticancer effects [9,10,11]. Additionally, it exhibits ameliorating effects in ischemic stroke [4], neuroprotective effects in ischemia reperfusion injury [7,8] and Parkinson’s disease [12], wound-healing properties [13], protective effects against kidney damage [6,14], and health-promoting effects on bone [15]. In particular, curcumin has shown vasodilatory and hypotensive effects. Curcumin was found to significantly improve the concentration-dependent vasodilatory effect of acetylcholine (ACh) and sodium nitroprusside (SNP) on the carotid artery in spontaneously hypertensive rats (SHRs) [16]. Curcumin also enhanced the vasorelaxant effect of ACh in the aortic ring of 2 K-1C-induced hypertensive rats [17]. Administrating curcumin dissolved in polyethylene glycol 400 (PGE400) to SHRs at 100 mg/kg/day for 3 weeks was found to remarkably reduce systolic blood pressure (SBP) by 27.1 ± 11.1 mmHg [18]. Curcumin dissolved in propylene glycol (PG) was found to lower SBP in a dose-dependent manner in 2K-1C-induced renovascular hypertension rats [17]. Therefore, curcumin is expected to be helpful in preventing or treating hypertension.

In their 2019 guidelines, the American College of Cardiology (ACC) and the American Heart Association (AHA) reported that hypertension leads to atherosclerotic cardiovascular disease (ASCVD) death more than any other modifiable ASCVD risk factor. Hypertension, which can be defined as SBP ≥ 130 mmHg or diastolic blood pressure of (DBP) ≥ 80 mmHg, has a prevalence of 46% in adults in the United States and rapidly increases with age [19]. Amlodipine, a widely used antihypertensive drug, relaxes smooth muscle in the heart and blood vessels by blocking the movement of calcium. When the smooth muscle relaxes, the afterload is eventually lowered, along with blood pressure [20]. Amlodipine should be administered daily at a dose of 2.5–10 mg/day [21]. According to the Medical Expenditure Panel Survey (MEPS) from to 2008–2018, the estimated number of prescriptions in the United States for amlodipine in 2018 was 75,811,947, making it the fifth mostly prescribed drug. The number of prescriptions for amlodipine showed an upward trend from 2008 to 2018 [22].

Since curcumin also relaxes blood vessels and lowers blood pressure, it might have beneficial effects when administered in combination with curcumin. The co-administration of curcumin and piperine, an alkaloid compound of black pepper (*P. nigrum*), was found to improve the bioavailability of curcumin [23], and the co-administration of curcumin and arabinogalactan was found to induce the apoptosis of breast cancer cells and inhibit tumor growth through p53 overexpression [24]. However, there have been no studies on the effects of the co-administration of curcumin and amlodipine on hypertension.

Amlodipine is a widely used antihypertensive drug that is ideally taken daily. As a widely used spice, many hypertensive patients who take amlodipine daily may also consume turmeric or curcumin for food and other medicinal purposes. However, there are no guidelines for patients with hypertension on taking amlodipine and curcumin together. Therefore, we sought to assess the effects of amlodipine and curcumin on blood pressure and aortic vessel relaxation in SHRs.

## 2. Materials and Methods

### 2.1. Chemicals and Reagents

Curcumin, amlodipine besylate, glucose, MgSO_4_, KH_2_PO_4_, KCl, NaCl, CaCl_2_, NaHCO_3_, phenylephrine (PE), and dimethyl sulfoxide (DMSO) were purchased from Sigma-Aldrich (St. Louis, MO, USA). Urethane and acetic acid were purchased from Daejung Chemicals & Metals Co., Ltd. (Siheung, Korea). HPLC-grade methanol, ethanol, acetonitrile, and water were purchased from J.T. Baker (Philipsburg, NJ, USA). All other reagents were of analytical purity. Curcumin and amlodipine were dissolved in DMSO, and the curcumin used in HPLC analysis was dissolved in methanol.

### 2.2. Animal

Male Sprague Dawley rats (SD; 240–260 g and 8 weeks old) were purchased from Daehan Biolink (Chungbuk Province, Korea). Male SHRs were purchased from Charles River Laboratories (Yokohama, Japan). All animal procedures were conducted according to the animal welfare guidelines and were approved (KHSASP-21-050) by the Kyung Hee University Institutional Animal Care and Use Committee. The rats were raised under standard laboratory conditions (22 ± 2 °C; lighting, 07:00–19:00), and food and water were provided ad libitum.

### 2.3. Plant Material and Extraction

**Figure 1:**
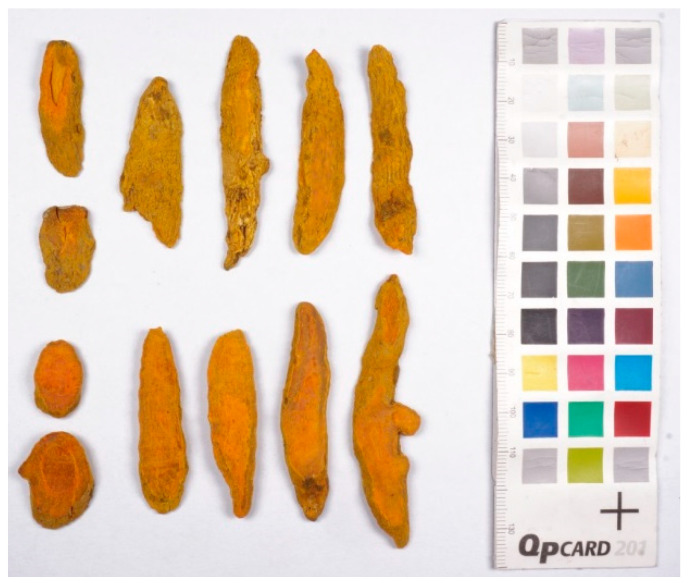
The appearance of *C. longa*.

*Curcuma longa* L. was purchased from Kwangmyungdang Medicinal Herbs (Ulsan Province, Korea) in June 2019 (Figure 1). The voucher specimen (number CL 001) of *C. longa* was deposited at the College of Korean Medicine, Kyung Hee University, Seoul, Republic of Korea. *C. longa* was ground using a blender machine and extracted using the reflux extraction method. The powder of *C. longa* (200 g) was extracted with 1 L of 30% (*v*/*v*) aqueous ethanol and water at 70 ± 5 °C for 3 h. The 30% ethanol and aqueous extracts were filtered with filter paper and concentrated in a rotary vacuum evaporator (N-N series, EYELA, Japan) at 50 °C. Subsequently, the concentrates were lyophilized in a freeze-dryer (Operon^TM^, Seoul, Korea) to obtain extracts (17.44 and 16.9 g, respectively). The lyophilized extracts were transferred into a vial and kept in a desiccator layered with silica gel for further use.

### 2.4. Preparation of Rat Aortic Rings

SD rats were anesthetized by the intraperitoneal injection of urethane (1.2 g/kg BW) before the isolation of the aorta. After the rats were completely anesthetized, the thoracic aorta was immediately removed and immersed in Krebs–Henseleit (KH) buffer (composition (mM): NaCl, 118.0; KCl, 4.7; MgSO_4_, 1.2; KH_2_PO_4_, 1.2; CaCl_2_, 2.5; NaHCO_3_, 25.0; and glucose, 11.1; pH 7.4) and then aerated with a gas mixture of 95% O_2_ and 5% CO_2_ at 37 °C. After carefully removing the connective tissue and fat surrounding the aorta, the aorta was cut into 2 mm long rings and suspended in organ chambers containing a 10 mL KH buffer at 37 °C. The rings were suspended between two tungsten stirrups, and one stirrup was connected to an isometric force transducer (Grass Instrument Co., Rhode Island, USA). The aortic ring segments were incubated under no tension for 15 min and left to equilibrate for 20 min at an optimal resting tension of 1.2 g. The KH buffer was refreshed 2 times during equilibration. The changes in tension of the aortic rings were recorded using isometric transducers connected to a data acquisition system (PowerLab, ADI instrument Co., New South Wales, Australia).

### 2.5. Measurement of Vascular Tension

To compare the vasorelaxant effect of *C. longa* extracts by extraction solvent, the aortic rings were pre-contracted with phenylephrine (PE, 1 μM) in a standard KH buffer. After an equilibration period, *C. longa* 30% ethanol and aqueous extracts were administered at cumulative concentrations (100, 200, 500, and 1000 μg/mL) to measure the relaxation effect of pre-constricted blood vessels. The extracts were administered after the tension in the blood vessel had plateaued.

To determine the optimal concentrations for the co-administration of curcumin with amlodipine, curcumin (10, 20, 50, and 100 μg/mL) and amlodipine (5 μg/mL) were administered to PE pre-contracted aortic rings.

The vasodilation effects of the co-administration of amlodipine and curcumin were measured by dividing the group into a amlodipine (5 μg/mL) single administration group and a curcumin (50 μg/mL) with amlodipine (5 μg/mL) co-administration group.

### 2.6. Measurement of Blood Pressure in SHRs

The SBP and DBP of SHRs were measured using the noninvasive tail cuff method (CODA 8-Channel High Throughput Non-Invasive Blood Pressure System, Kent Scientific Co. Ltd., Torrington, CT, USA) [25]. To evaluate the hypotensive effect of the co-administration of amlodipine and curcumin, the experimental groups were divided into four groups: an amlodipine (1 mg/kg, i.p.) single administration group and three curcumin (30, 100, or 300 mg/kg, i.p.) with amlodipine (1 mg/kg, i.p.) co-administration groups. The 24 SHRs were randomly divided into four groups of six animals each.

SHRs were acclimated for one week, and their blood pressure was measured once daily during the adaptation period. SHRs were allow to acclimatize inside the restraint for 10 min before the blood pressure measurement was started. The changes in blood pressure were calculated by subtracting the blood pressure prior to administration from the blood pressure recorded at each hourly measurement. The SBP and DBP of SHRs were measured before administration and at 1, 2, 4, and 8 h after drug administration.

### 2.7. Curcumin Content of Different Solvent Extracts from C. longa by HPLC Analysis

A Waters e2695 Alliance HPLC system (Waters Corp., Milford, MA, USA) connected to a photodiode array detector (PDA) 2998 and Empower 2 software was used for the quantitative analysis. Chromatographic separation was performed on a Sunfire^®^C18 reversed-phase column (4.6 × 250 mm, 5 μm) (Waters Corp., Milford, MA, USA), with column oven temperature maintained at 25 °C. One gram of *C. longa* in 30% ethanol and aqueous extracts were dissolved in 10 mL of HPLC-grade methanol. The extract was filtered through a 0.45 µm polyvinylidene difluoride syringe filter (Macherey-Nagel, Düren, Germany). The mobile phase consisted of 0.1% acetic acid (solvent A) and acetonitrile (solvent B) at a flow rate of 1.0 mL/min. The percentage composition of solvent B was as follows: 35% at 0–15 min and 35–100% at 15–20 min. The injection volume was 10 μL, and all sample analyses were performed in triplicate. UV absorbance was monitored at 420 nm. The quantity of curcumin in *C. longa* extracts was calculated as follows: the amount (mg) of standard material = the quantitative amount (mg) of standard materials × AT/AS/n (*n* = 3; AT = the peak area of the test sample containing the standard; AS = the peak area of the standard). The percentage composition of curcumin in the extracts was calculated from the HPLC peak areas.

### 2.8. Statistical Analysis

SPSS (version 21.0) statistical analysis software (SPSS Inc., Chicago, IL, USA) and GraphPad Prism 5 software (San Diego, CA, USA) were used for the statistical analysis of the data. Data passed the Kolmogorov–Smirnov (KS) normality test. Results are expressed as the mean ± standard error of the mean (SEM). Statistical comparisons were made using one-way ANOVA, two-way ANOVA, and the independent samples *t*-test. Statistical significance was set at *p* < 0.05.

## 3. Results

### 3.1. Vasorelaxant Effect of Different Extraction Solvents of C. longa

**Figure 2:**
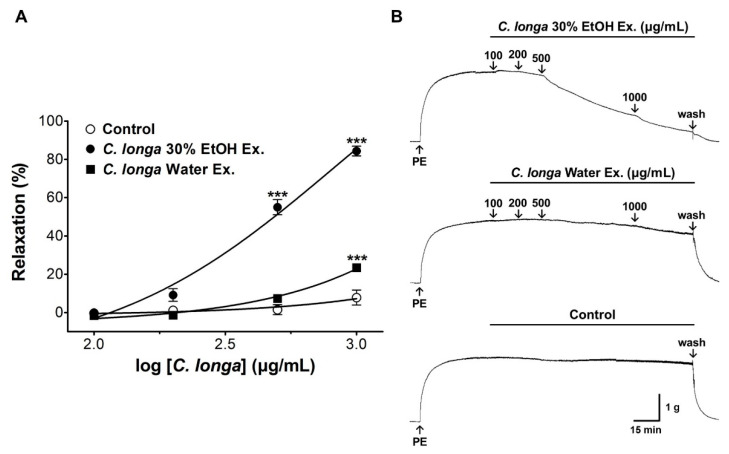
Cumulative concentration–response curve for each extraction solvent of *C. longa* extracts (100–1000 μg/mL) in rat aortic rings pre-contracted with phenylephrine (PE, 1 μM) (**A**). Representative traces under the indicated conditions (**B**). Values are expressed as mean ± SEM (*n* = 6). An unpaired *t*-test was used for statistical comparisons. *** *p* < 0.001 vs. control.

The 30% ethanol and aqueous extracts of *C. longa* showed vasorelaxant effects in rat aortic rings pre-contracted with PE (1 μM). The vasorelaxant effects of the 30% ethanol and aqueous extracts of *C. longa* were 55.0 ± 3.9% and 7.3 ± 1.0% at 500 μg/mL and 84.3 ± 2.5% and 23.5 ± 1.9% at 1000 μg/mL, respectively (Figure 2).

### 3.2. Curcumin Content of Different Solvent Extracts from C. longa by HPLC Analysis

**Figure 3:**
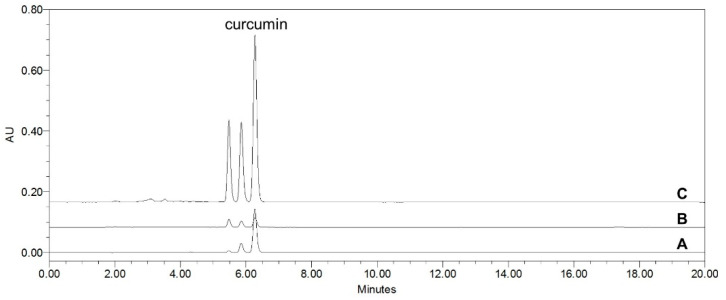
HPLC chromatogram of curcumin standard solution (A), *C. longa* aqueous extract (B), and *C. longa* 30% EtOH extract (C) at 420 nm.

The retention time of curcumin was 6.27 min. The standard curve was calibrated using linear regression derived from the peak area. The regression equation (correlation coefficient, R^2^) of curcumin was y = 77131.75x + 169542.17 (0.9998), which exhibited good linearity. The curcumin contents of the *C. longa* 30% and aqueous extracts were 2.64 ± 0.01% and 0.05 ± 0.00%, respectively (Figure 3).

### 3.3. Vasorelaxant Effect of Curcumin

**Figure 4:**
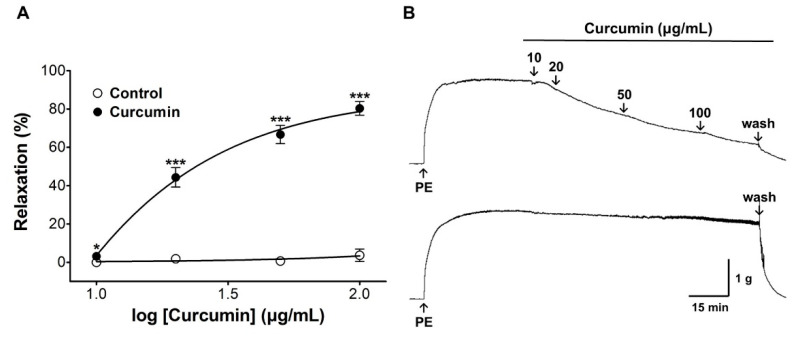
Cumulative concentration–response curve of curcumin (10–100 μg/mL) in rat aortic rings pre-contracted with phenylephrine (PE, 1 μM) (**A**). Representative traces under the indicated conditions (**B**). Values are expressed as mean ± SEM (*n* = 6). An unpaired *t*-test was used for statistical comparisons. * *p* < 0.05 and *** *p* < 0.001 vs. control.

The vasorelaxant effects of curcumin were 44.3 ± 5.1% at 20 μg/mL, 66.7% ± 4.7% at 50 μg/mL, and 80.3 ± 3.6% at 100 μg/mL (Figure 4). Curcumin caused vasorelaxation in aortic rings pre-contracted with PE (1 μM) in a concentration-dependent manner.

### 3.4. Vasorelaxant Effect of Co-Administration of Curcumin and Amlodipine

**Figure 5:**
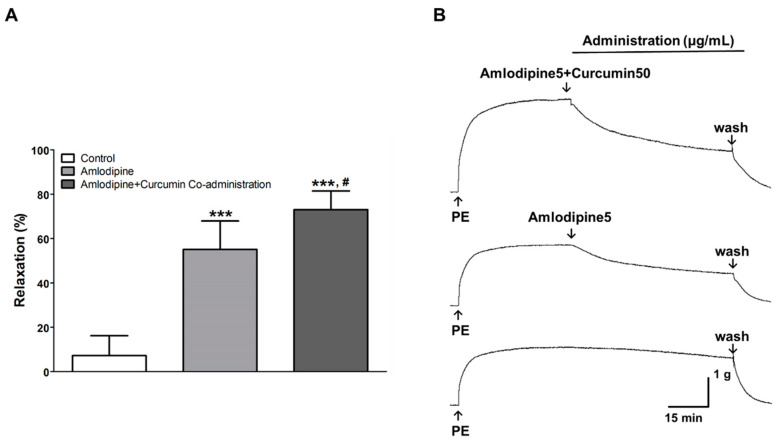
Vasorelaxant effect of the single administration of amlodipine (5 μg/mL) and the co-administration of amlodipine (5 μg/mL) and curcumin (50 μg/mL) in rat aortic rings pre-contracted with phenylephrine (PE, 1 μM) (**A**). Representative traces under the indicated conditions (**B**). Values are expressed as mean ± SD (*n* = 6). The one-way ANOVA was used for statistical comparisons. *** *p* < 0.001 vs. control and ^#^ *p* < 0.05 vs. amlodipine.

To evaluate the effect of the co-administration of amlodipine and curcumin, a dose that relaxes the blood vessels by approximately 50% was selected as the co-administration dose. The co-administration of curcumin (50 μg/mL) with amlodipine (5 μg/mL) showed a higher vasorelaxant effect than the administration of amlodipine alone. When amlodipine was administered alone, the maximal vasodilatory effect was 55.0 ± 12.9% (95% CI: 41.5–68.5%); in comparison, the effect was 73.0 ± 8.4% (95% CI: 64.2–81.8%) when curcumin and amlodipine were co-administered (Figure 5).

### 3.5. Hypotensive Effect of Co-Administration of Curcumin and Amlodipine

**Figure 6:**
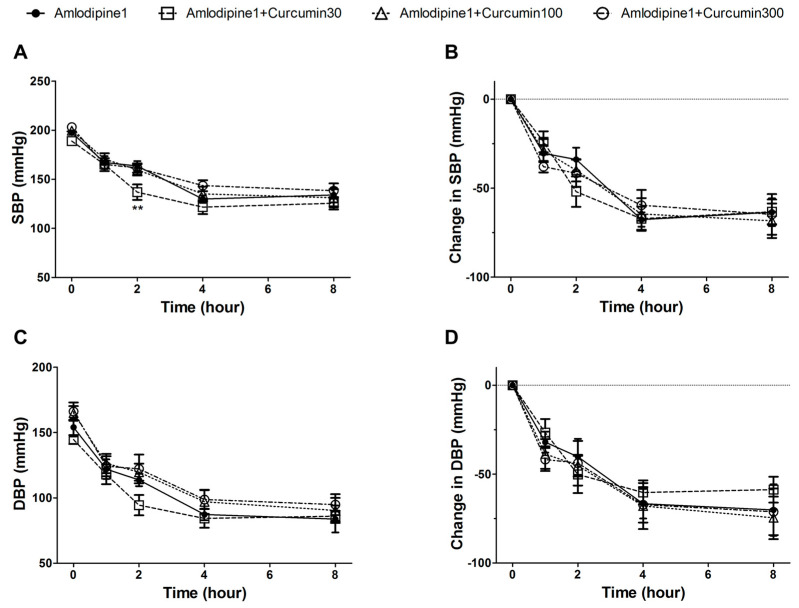
Systolic blood pressure (SBP) (**A**), change in SBP (**B**), diastolic blood pressure (DBP) (**C**), change in DBP (**D**) measured in spontaneously hypertensive rats (SHRs) at baseline and 1, 2, 4, and 8 h after the single administration of 1 mg/kg of amlodipine (●) and the co-administration of 1 mg/kg of amlodipine and 30 mg/kg (□), 100 mg/kg (△), and 300 mg/kg (○) of curcumin. Values are expressed as mean ± SEM (*n* = 6). The two-way ANOVA was used for statistical comparisons. ** p < 0.01 vs. control.

At the beginning of treatment, the average SBP values in the curcumin (30, 100, and 300 mg/kg) with amlodipine (1 mg/kg) co-administered groups and the amlodipine single administrated group (control) were similar (197.5 ± 2.5, 188.8 ± 1.8, 199.5 ± 3.3, and 203.1 ± 4.4 mmHg, respectively). The average DBP values were measured to be 154.0 ± 7.0, 144.6 ± 3.4, 164.9 ± 5.2, and 166.0 ± 6.9 mmHg, respectively. The SBP and DBP in all groups decreased steadily until 4 h after administration and remained similar until 8 h. At 2 h after administration, a significant SBP value was observed in the 30 mg/kg curcumin co-administered group but not in the value of change in SBP. There was no significant difference between the other groups. The control group and the co-administration group altered SBP by −63.5 ± 7.8, −63.3 ± 6.9, −68.3 ± 9.7, and −64.8 ± 11.4 mmHg at 8 h after treatment, and the alternation of DBP was measured to be −70.2 ± 14.5, −58.7 ± 7.3, −74.6 ± 12.0, and −71.2 ± 13.1 mmHg, respectively (Figure 6).

## 4. Discussion

In the present study, the vasorelaxant effects of aqueous and 30% ethanol extracts of *C. longa*, curcumin, amlodipine besylate, and curcumin co-administered with amlodipine—as well as the hypotensive effect of the co-administration of curcumin and amlodipine—were evaluated. Both the *C. longa* 30% ethanol and aqueous extracts showed vasodilatory effects in the rat thoracic aorta pre-contracted with PE. At the same concentration of 1000 μg/mL, the aqueous and 30% ethanol extracts induced vasorelaxation by 23.5 ± 1.9% and 84.3 ± 2.5%, respectively. These results suggest that the vasorelaxant effect of *C. longa* is mostly due to the components of *C. longa* extracted into organic solvents.

Curcumin is the main active compound in turmeric [26]. In the present study, the contents of curcumin in the 30% ethanol and aqueous extracts were 2.64 ± 0.01% and 0.05 ± 0.00%, respectively. Curcumin showed a concentration-dependent vasodilatory effect in isolated rat thoracic aortic rings. Curcumin content in turmeric extracted using the high hydrostatic pressure extraction (HHPE) method was found to depend on the concentration of ethanol in the extractant. As the concentration of ethanol increased, curcumin was detected to be 5.31 ± 0.06 and 9.65 ± 0.05 μg/g at 0% and 20% ethanol, respectively, in the extraction solvent [27]. The contents of other curcuminoids, bisdemethoxycurcumin and demethoxycurcumin, also increased as the concentration of ethanol increased. Bisdemethoxycurcumin and demethoxycurcumin were only detected in more than 70% and 40% ethanol concentrations, respectively [27]. The methanol extract of turmeric was found to exhibit endothelium-independent vasorelaxant effects by inhibiting extracellular and intracellular Ca^2+^ in the porcine basilar artery pre-contracted with U46619 [28]. Curcumin promotes the endothelium-dependent vasodilatory response of SNPs by inducing nitric oxide (NO) release [16]. Curcumin was found to improve endothelial dysfunction by increasing NO bioavailability and decreasing levels of the angiotensin-converting enzymes MMP-2 and MMP-9 in hypertensive rats [17]. Therefore, the 30% *C. longa* extract having better vasodilatory effects than the aqueous extract can be attributed to its higher curcumin content.

Many antihypertensive agents exhibit hypotensive effects by decreasing the cardiac output or peripheral resistance of blood vessels. These antihypertensive agents include diuretics, Ca^2+^ channel blockers, angiotensin-converting enzyme inhibitors, angiotensin receptor blockers, and renin inhibitors. Ca^2+^ channel blockers are generally used when primary drugs for the treatment of hypertension in patients with diabetes or hypertension are ineffective [29]. Amlodipine is an oral dihydridine calcium channel blocker administered once a day because of its long half-life of 30–50 h [30]. Amlodipine is used as a single drug, but it is also used in co-administration with other drugs to achieve a synergistic effect. Atorvastatin [31], a lipid-lowering agent that blocks cholesterol synthesis; aliskiren [32], an inhibitor that reduces the activation of the renin-angiotensin aldosterone system (RAAS); hydrochlorothiazide [32], a thiazide diuretic; benazepril and perindopril [33], angiotensin-converting enzyme inhibitors; and olmesartan, telmisartan, and valsartan [34], the angiotensin-II receptor blockers; are commonly used in combination with amlodipine. In addition, cheonwangbosimdan, a traditional herbal medicine, was co-administered with amlodipine and showed high vasodilatory and blood pressure-lowering effects [35].

Many hypertensive patients taking amlodipine often consume turmeric or curcumin for food or as herbal medicine. However, there is no research on the co-administration of curcumin and amlodipine for vasodilation or lowering blood pressure. In the present study, amlodipine (5 μg/mL) relaxed the rat thoracic aortic rings pre-contracted with PE to 55.0 ± 12.9%. On the other hand, the co-administration of amlodipine (5 μg/mL) with curcumin (50 μg/mL) relaxed the pre-contracted aortic rings to 73.0 ± 8.4%. However, as a result of evaluating the effect on blood pressure using SHRs, no significant difference was found between the co-administration of amlodipine (1 mg/kg) with curcumin (30, 100, and 300 mg/kg) and the administration of amlodipine (1 mg/kg) alone. Though blood pressure was significantly lowered 2 h after the co-administration of curcumin (30 mg/kg) and amlodipine (30 mg/kg), compared to amlodipine (1 mg/kg) alone, no significant decreases were observed in other SBP or DBP values up to 8 h. In addition, the change in SBP value did not show any significance even at 2 h, and no dose-dependent effects were observed. Therefore, it was thought to be a statistical error caused by the difference in the baseline blood pressure values of the individual. As such, the effect of co-administration was better in ex vivo, but the reason there were no differences in in vivo was presumed to be the poor bioavailability of curcumin due to its poor absorption, rapid systemic elimination [36], and high rate of metabolism [23].

No side effects were observed after the co-administration of curcumin and amlodipine. The maximum dose of curcumin used in the in vivo experiment (300 mg/kg) was equivalent to 600 g/kg of the *C. longa* aqueous extract and 11 g/kg of the *C. longa* 30% ethanol extract. Various studies have reported the safety of curcumin dosage. Curcumin is a compound with proven safety, and it is non-toxic to humans even if taken orally at 8 g/day for 3 months [37]. Short- or long-term administration (13 weeks or 2 years) to rats at doses up to 2600 mg/kg did not show toxicity in either male or female rats [38]. According to the National Cancer Institute, the administration of 3.5 g/kg for 3 months did not exhibit toxic effects in rats, dogs, and monkeys [39]. In phase I safety trials, the daily intake of curcumin (12 g) was also shown to be safe [40]. According to The Korea National Health and Nutrition Examination Survey of 2008–2012, Koreans consumed 0.47 g/day of turmeric per day. [41]. In a toxicity evaluation experiment of a curcuminoid–essential oil complex (CEC) containing 95% curcuminoid, CEC showed no toxicity even at a dose of 5000 mg/kg [42], and a turmeric extract formulation (comprising turmeric) that was developed to increase bioavailability showed no side effects even at 3000 mg/kg of administration [43]. Therefore, our results suggest that the co-administration of curcumin or turmeric with amlodipine is safe and without side effects.

In conclusion, although the co-administration of curcumin and amlodipine did not show a synergistic effect on lowering blood pressure, the results of this study suggest that intake of curcumin or turmeric while taking amlodipine did not augment the blood pressure-lowering effect of amlodipine.

## Data Availability

The data presented in this study are available on request from the corresponding author.
